# Reduction in muscular motility by selective focused cold therapy: a preclinical study

**DOI:** 10.1007/s00702-013-1077-y

**Published:** 2013-08-06

**Authors:** Michael Hsu, Fang-Feng Stevenson

**Affiliations:** Myoscience, Inc, 1600 Seaport Blvd, Suite 450, Redwood City, CA 94063 USA

**Keywords:** Cryotherapy, Cryosurgery, Cold temperature, Wrinkle, Botulinum toxin, Aesthetic

## Abstract

Application of freezing temperatures to the temporal branch of the facial nerve can temporarily inhibit motor nerve conduction, resulting in inhibition of voluntary contraction of the frontalis and glabella muscle groups. This feasibility study demonstrates the reduction in motility of muscle groups through application of low temperatures to nerves in a rat model. Twenty-seven adult female Sprague–Dawley rats received cryotreatment to the tibial nerve of the hind limb, and the contralateral limb was left untreated as a negative control. The use of a cold temperature application (−59 ± 8 °C for 60 s) onto the rat tibial nerve resulted in temporary reduction of physiological function of the hind limb. Histological observations of the nerve revealed demyelination and axonal degeneration by 2 weeks post-treatment followed by complete axonal regeneration and remyelination at 16 weeks. Application of low temperatures to peripheral motor nerves resulted in temporary denervation and loss of function of the treated hind limb. Low temperature treatment on motor nerves did not result in any permanent or long-term changes to function and structure of the nerves.

## Introduction

Cryosurgery uses freezing temperatures to either disrupt tissue function or ablate unwanted tissue. The basic mechanism of action in the freezing of tissue is the formation of ice crystals which results in the disruption of cell membrane and its organelles. Cells exposed to freezing temperatures of about −5 to −15 °C at the periphery of the ice crystal/ice ball primarily undergo apoptotic cell death, whereas colder temperatures at the center of the cell leads to necrotic instead of apoptotic cell death (Jackson et al. 2005; Tatsutani et al. 2005). Over time, necrotic cell death occurs at colder temperatures (<−20 °C) because intracellular ice formation directly damages cell organelles and membranes. At the periphery where the temperatures are warmer (−20 to 0 °C), ice formation primarily occurs extracellularly. The extracellular ice formation causes the environment to become hyperosmotic, thus removing water from the cells. Drastic osmotic changes within a  cell leads to severe cellular dysfunction resulting in apoptotic cell death (Pegg 2010).

The local application of low temperatures has been used as a medical therapy for centuries before being adapted by modern medicine (Cooper and Dawber 2001). Early use of cold occurred over a small range of temperatures (+10 to −5 °C) and treatment resulted in the temporary inactivation of the nerve (neuropraxia), which produced a nerve conduction block that could last from several hours to days (Rosenberg and Heavner 1980; Gage et al. 2009). Since then, cryosurgery has become the most common advanced use of cold for medical treatment. Though this method is well established and has been proven safe through decades of clinical use, it was not considered an ideal method for the treatment of motor or sensory nerves due to the extreme low temperatures used during treatment (~−180 °C). With new technological advancements, it has become possible to treat tissue over more specific temperature ranges, allowing for the treatment of sensory or motor nerves, known generically as cryoanalgesia and cryoneuromodulation, respectively.

Focused cold therapy (FCT) is the direct application of low temperatures to inhibit signaling of peripheral nerves. Exposure to temperatures between −20 and −88 °C leads to axonal and myelin degeneration, also known as Wallerian degeneration (Campos et al. 2009). Morphologically, this process is characterized by a beading appearance followed by granular disintegration of the axons at and distal to the site of exposure (Feng et al. 2010). In Wallerian degeneration, the acellular nerve structures such as the endoneurium, perineurium and epineurium are preserved, allowing normal axonal regeneration and remyelination (Moorjani et al. 2001). This is in contrast to more traumatic nerve injuries (i.e., transection or thermal heat lesions), which disrupt the acellular nerve structures, sometimes causing neuroma formation and aberrant axon regeneration (Campos et al. 2009).

Previous studies have assessed the recovery of the peripheral nerves following cryogenic injury and detail the behavioral, electrophysiological and pathological recovery of these peripheral nerves after exposure to temperatures as low as −120 °C (Barnard 1980; Willenbring et al. 1995; Popken et al. 2003; Jiang et al. 2010). However, these studies focus on the structural and functional recovery of sensory nerves; there have been very few studies performed to identify the effects of FCT of peripheral motor nerves. In particular, there has been a lack of studies demonstrating long-term histological and physiological observations after multiple treatments.

FCT can be used on motor nerves for various medical conditions, including but not limited to: movement disorders, muscle spasms, muscle hyperactivity and/or any condition where reduction in muscle movement is desired. This paper will present a rat nerve model and demonstrate a temporary loss of motor function via the application of a cryotreatment on the sciatic nerve of the hind limb. The physiological and histological changes up to 16 weeks after treatment will be presented.

## Methods and materials

Animal studies were performed at Stanford animal facilities (Palo Alto, CA, USA), and the study protocol was approved by the Stanford Institutional Animal Care and Use Committee. Twenty-seven adult female Sprague–Dawley rats (250–300 g) were housed two to a cage with standard chow and water available ad libitum. They were maintained on a 12-h light cycle for the duration of the study.

**Fig. 1:**
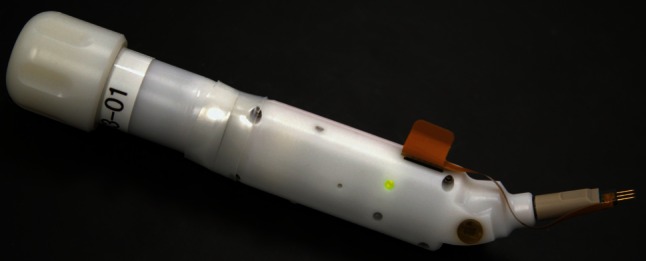
Myoscience Cryo-Touch II Device

The Myoscience Cryo-Touch II Device (Redwood City, CA, USA) was used to apply FCT to the nerves (Fig. 1). This handheld device delivers compressed liquid nitrous oxide to a close-ended 27 gauge, 6 mm long three needle probe (trident probe). A type T, bifilar 0.0015″ thermocouple was soldered on the tip of a cryoprobe to monitor treatment temperatures.

During the studies, animals were surgically treated with the device on the tibial nerve of the left leg. The right leg was left untreated as the contralateral control. The animals were placed under general anesthesia with a mixture of 2.5 l/min O_2_ and 2.5 % isoflurane. Under aseptic conditions, the sciatic nerve was surgically accessed through the thigh targeting the trifurcation point (tibial, sural and peroneal nerves). The tibial nerve was visually identified and the cryoprobe placed in direct contact with the nerve. The device applied a 1 min treatment at −56 °C (−55.9 °C average; range −51 to −61 °C). After treatment, the nerve was returned to the host bed and the incision was closed using standard two-layer technique. The animal was allowed to recover from the anesthesia and then returned to its standard housing.

Physiological toe spread and motor function were assessed twice a week until return to normal function was observed. Untreated contralateral hind limb was measured as negative control.

A modified toe-spread test was performed to determine the change in muscle function (Varejão et al. 2004). Toe spread was determined by lifting the rat by the base of the tail with the legs hanging freely. The toe-spread reflex was observed with the following ratings: normal reflex with all toes spreading out received a score of 0; weak reflex with some toes partially spreading apart received a score of 1; and lack of reflex with all toes clubbed together received a score of 2.

Motor function was measured by trained laboratory personnel who were blinded to the animal study group. These reviewers assessed the walking behavior of the animal and assigned a score (Brummett et al. 2008). The motor function was observed with the following ratings: normal walking function received a score of 0; normal dorsiflexion ability while walking with curled toes received 1; moderate dorsiflexion ability while walking with curled toes received and no dorsiflexion ability while walking with curled toes received 3.

The treated nerves were dissected at 2, 8 and 16 weeks post-treatment and submersed in 10 % neutral buffered formalin. Tissues were embedded in paraffin, sectioned and placed on slides (standard technique). General morphological assessment was performed by staining the tissue sections with hematoxylin and eosin stains.

Separate paraffin nerve sections from the same specimens were also subsequently stained for immunological assessment for neurofilament (NF) and S-100 expression. Standard immunofluorescence technique was performed with the following specific details for this assay. The double staining was performed sequentially, starting with the S-100 staining. Incubation with rabbit polyclonal S-100 (Abcam, #ab34686) was followed with goat anti-rabbit Alexa Fluor 586 (Invitrogen) as the secondary antibody. Next, mouse monoclonal NF-M (Santa Cruz Biotechnologies, clone: 3H11) was applied and followed with goat anti-mouse Alexa Fluor 488 as the secondary antibody. 4′,6-Diamidino-2-phenylindole (DAPI; Sigma) was employed to stain nuclei. One percent BSA in 1× PBS with 0.05 % Tween-20 was used as the staining buffer. Immunostaining was analyzed with a fluorescence microscope (Zeiss Axioskop). Axon density was manually counted for NF positive stained axons in seven imaging fields per time point.

## Results

**Fig. 2:**
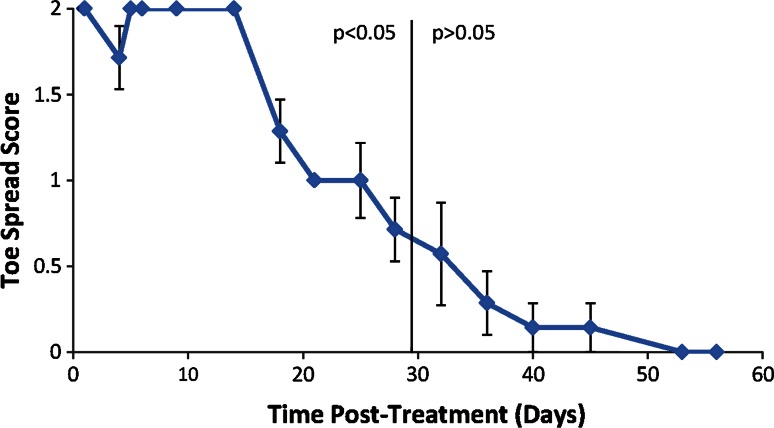
Toe-spread score of animals receiving FCT at the tibial nerve (*n* = 10). *Error bars* shown represent 1 SD. When no *error bars* are shown, it indicates that the standard deviation is zero

Measurement of toe-spread ability was used to quantify the weakening of the treated hind limb as compared to the contralateral control (Fig. 2). All animals demonstrated lack of toe spread with a score of 2 starting at 1 day post-treatment. Strong weakening of the toe-spread function continued for 14 days until gradual return to normal toe function by about 53 days post-treatment. Significant difference between the treated and control legs was seen for 28 days (*p* < 0.05, calculated as compared to the contralateral untreated control.). No abnormal or dysfunctional movements were observed after complete functional recovery. Untreated control legs demonstrated a score of 0 throughout the post-treatment assessments (not shown).

**Fig. 3:**
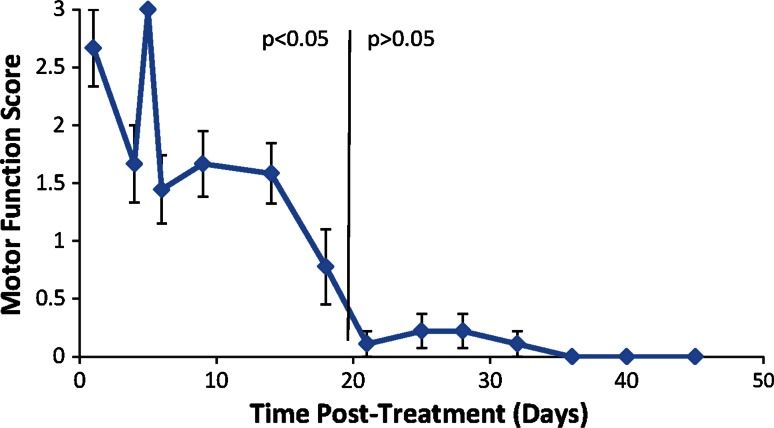
Motor function assay from animals that received treatment at the tibial nerve (*n* = 10). *Error bars* shown represent 1 SD. When no *error bars* are shown, it indicates that the standard deviation is zero

The motor function (walking ability) assay measured the loss of function of the hind limb after cryotreatment. Motor function weakening was demonstrated with an average score of 2.7 ± 0.3 in the treated leg starting at 1 day post-treatment (Fig. 3). Gradual return to normal walking ability was seen by 36 days post-treatment. Significant difference between the treated and control legs was noted for 18 days (*p* < 0.05, calculated as compared to the contralateral untreated control). No abnormal or dysfunctional walking ability was observed after full recovery in hind limb function. Untreated control legs demonstrated a score of 0 throughout the post-treatment assessments (not shown).

**Fig. 4:**
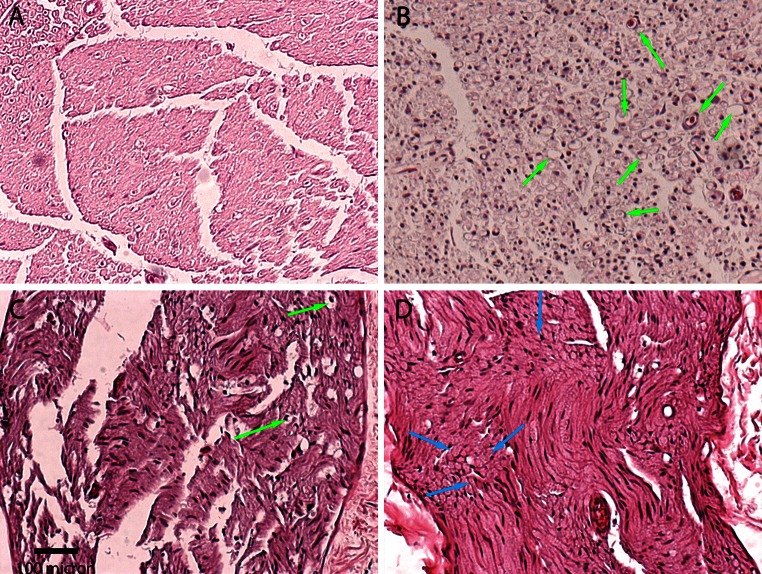
Cross sections of H&E-stained nerves were assessed at **b** 2, **c** 8 and **d** 16 weeks post-treatment. **a** Untreated control was used for comparison. *Green arrow* indicates degenerated axons (vacuolation). *Blue arrow* indicates regenerated axon

Histological assessments were performed on the treated nerves at 2, 8 and 16 weeks post-treatment (Fig. 4). Axonal degeneration and neural disruption were observed at the 2-week time point. Vacuolations were observed throughout the cross section (green arrows), indicating degeneration and loss of axons. Few degenerating axons could be identified by the punctated staining within the vacuolated areas. Condensation of the myelin sheath was observed, while the basal laminae remained intact at this time point. The presence of macrophage population was observed.

At 8 weeks post-treatment, significant axonal regeneration with concurrent elimination of macrophage infiltration was observed. The newly regenerated axons appeared re-myelinated, while few degenerative axon fibers were noted. Few vacuolated areas were persisting at this time point.

At 16 weeks post-treatment, samples appeared normal. There were no vacuolations observed and axons appeared fully regenerated (blue arrows). No observable occurrences of neuromas and/or fibrosis were observed in any of the samples.

**Fig. 5:**
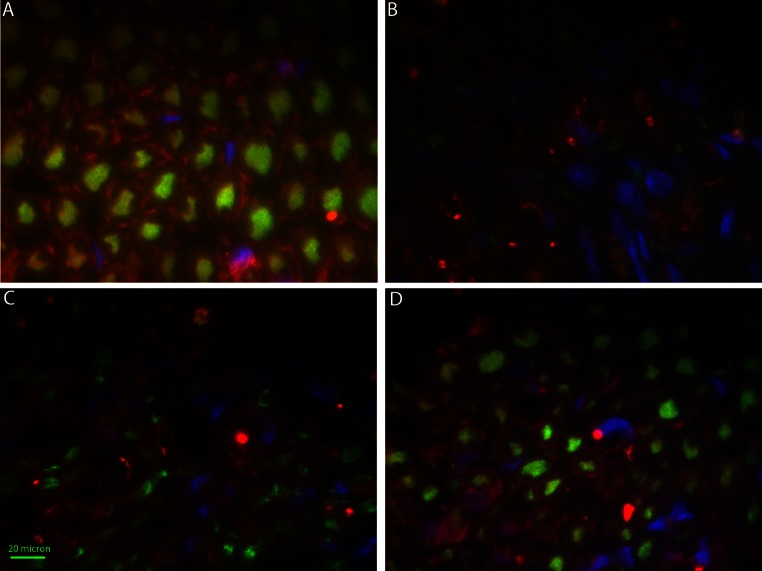
Immunofluorescently stained cross section of **a** untreated control, **b** 2 weeks, **c** 4 weeks and **d** 8 weeks post-treated nerves. *Green stain* indicates positive for neurofilament, *red stain* indicates positive for S100, and *blue stain* indicates nuclei

Staining for NF (axons) and S100 (Schwann cells) showed analogous patterns of degeneration and regeneration as the H&E histology (Fig. 5). NF (green), S100 (red) and DAPI (blue) were used to stain for axons, Schwann cells and nuclei (respectively). At 2 weeks, significant loss of NF-positively stained axons were observed. Punctate staining and reduction in S100-positively stained Schwann cells were observed. Increased nuclei population density was also noted.

At 8 weeks post-treatment, early regeneration of axons was noticeable by the presence of positively stained axons for NF. Early regeneration of axons was observed by the presence of a small size and low-density population of stained cells. Few punctate staining for S100 was present, but there was more diffuse staining throughout the section.

At 16 weeks, complete regeneration of the treated nerve was noted with NF positively stained axons delimited by the S100 positively stained Schwann cell. Further maturation of the axons was indicated by the increase in size of the axon diameter from the 8-week post-treated samples.

**Fig. 6:**
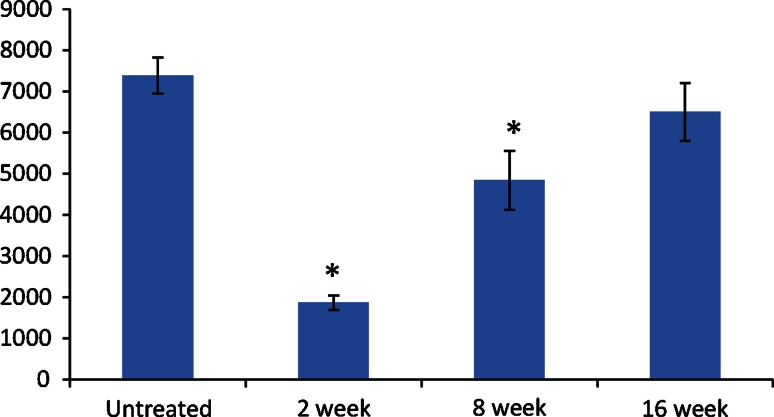
Axon densities were compared in untreated and 2, 8 and 16 weeks post-cryotreatment. *p < 0.05 as compared to untreated control. Counts were measured in axons/mm^2^

A count of axon density showed a significant reduction 2 weeks post-treatment followed by a gradual increase back to pre-treatment densities (Fig. 6). Axon density was measured as follows: untreated, 7,381 ± 439 axons/mm^2^; 2 weeks post-treatment, 1,872 ± 176 axons/mm^2^; 8 weeks post-treatment, 4,836 ± 703 axons/mm^2^; and 16 weeks post-treatment, 6,501 ± 531 axons/mm^2^. Axon density at 2 and 8 weeks post-treatment showed significant (*p* < 0.05) difference from control, while at 16 weeks post-treatment it showed no difference (*p* > 0.05) compared to untreated control (Fig. 6).

## Discussions/conclusions

The present study demonstrated that the direct application of freezing temperature (−56 °C) on a motor nerve will result in a temporary interruption of the target muscle function. The observed histological changes indicate second degree Wallerian degeneration as the mechanism of action. Wallerian degeneration was indicated by the loss of axons observed in both H&E and immunofluorescence loss of axons and reduced staining of S100. This demonstrated equivalent histological results as seen with historical studies using various freezing temperatures (Kerns et al. 1991). Histological analyses suggested that the axons and Schwann cells became disrupted, while the injury left the epineurium and perineurium intact. Use of freezing temperatures leaves the basal laminae of the endoneurium unperturbed. The Schwann cells are able to form the Bands of Büngner along the endoneurial tube, which aid in guiding regenerating axons to their target tissue. Immunofluorescence staining demonstrated gradual axon regeneration and re-myelination. Axon density measurements showed a return to normal levels in this study.

The temporary loss of axons was reflected in the functional loss of the treated hind limb. Disruption of the hind limb nerve conduction resulted in a deficit in toe-spread function and walking gait (see Figs. 2, 3). Toe-spread data showed increased weakening and longer duration compared to the walking motor function data. This difference arises from the different muscle groups being measured in each of these assays. Toe function relies on the function of the more distal muscle groups of the hind limb (i.e., extensor digitorum longus and digiti minimi abductor), while the motor function relies on both the distal and proximal muscle groups of the hind limb. This difference in motor function and toe-spread data reflects the focused nature of the treatment to the targeted nerve.

Full axonal regeneration was confirmed with complete restoration of axon density and morphological recovery comparable to the untreated nerve (see Fig. 4). Complete physiological function did not necessitate complete axonal regeneration. At 8 weeks, normal function was observed coincident with a 34 % decrease in level of normal axonal density (see Fig. 5c) relative to the untreated control (Fig. 5a). Motor function and toe-spread assay returned to normal by 8 weeks. There is sufficient axonal signaling to the target muscle groups to restore normal movement without complete axonal regeneration. The physiological assays currently used in this study did not capture the strength of the treated hind limb. A more elaborate study should further reveal the correlation between axon density, muscle tetanic forces and physiological function.

This proposed method of cooling a motor nerve to induce a temporary injury involving second degree Wallerian degeneration and reinnervation to reduce muscle contraction can be used in the clinical setting. In particular, this technology can be used to reduce dynamic facial wrinkles with FCT to specific facial motor nerve branches. Further use of this platform technology can expand to other indications, including movement disorders which involve hyperactive muscle functions such as dystonia, spasms and general weakening of a muscle control. Some of the current treatments for these types of movement disorders involve the use of neurotoxins and drugs which inhibit the nerve signaling to the muscle, sometimes with unwanted side effects. FCT treatment of the motor nerve can potentially be used as a drug-free method of relaxing the specific target muscle. Reduction of muscle function with the use of FCT is shown to be temporary followed by a normal return of function. Future studies will further elucidate the mechanisms of action that enable this behavior.
